# Fabrication of silver nanoisland films by pulsed laser deposition for surface-enhanced Raman spectroscopy

**DOI:** 10.3762/bjnano.10.89

**Published:** 2019-04-16

**Authors:** Bogusław Budner, Mariusz Kuźma, Barbara Nasiłowska, Bartosz Bartosewicz, Malwina Liszewska, Bartłomiej J Jankiewicz

**Affiliations:** 1 Institute of Optoelectronics, Military University of Technology, gen. W. Urbanowicza 2 Str. 00-908 Warsaw, Poland

**Keywords:** nanofabrication, pulsed laser deposition, SERS substrates, silver nanoisland films, surface-enhanced Raman spectroscopy, X-ray photoelectron spectroscopy

## Abstract

The results of studies on the fabrication and characterization of silver nanoisland films (SNIFs) using pulsed laser deposition (PLD) and the evaluation of these films as potential surface-enhanced Raman scattering (SERS) substrates are reported. The SNIFs with thicknesses in a range of 4.7 ± 0.2 nm to 143.2 ± 0.2 nm were deposited under different conditions on silicon substrates. Size and morphology of the fabricated silver nanoislands mainly depend on the substrate temperature, and number and energy of the laser pulses. SERS properties of the fabricated films were evaluated by measuring SERS spectra of *para*-mercaptoaniline (pMA) molecules adsorbed on them. SERS enhancement factors are shown to depend on the SNIF morphology, which is modified by changes of the deposition conditions. The highest enhancement factor in the range of 10^5^ was achieved for SNIFs that have oval and circular silver nanoislands with small distances between them.

## Introduction

In recent years, SERS has been intensively investigated as a sensing tool in many applications [1–3]. Of particular interest is the use of SERS as a method for highly sensitive detection of hazardous materials such as chemical and biological agents or explosive materials [4–5]. However, despite many studies SERS remains mainly a laboratory technique. To bring it closer to real-life applications there is a need to develop cheap, reliable, reproducible and efficient SERS substrates. The SERS effect is generally assumed to mainly originate in the electromagnetic field enhancement caused by a localized surface plasmon excitation in nanostructures through the incident laser light. With respect to the substrate. It depends on the size, shape, and arrangements of nanostructures, the material they are made of and the surrounding medium [6]. One of the easiest nanostructures to produce are metallic nanoparticles (NPs). Alone or in composites with other materials, they find numerous applications in plasmonic photocatalysis [7–9], photovoltaics [10] or optical sensing through localized surface plasmon resonance (LSPR) [11]. It is therefore not surprising that quite a number of studies have been initiated and performed in order to design and fabricate highly active SERS substrates based on metallic nanoparticles and nanoparticle films [12–14]. Metallic NPs of different sizes and shapes are prepared in solution mainly by chemical synthesis using various reducing agents and conditions [13]. However, physical methods such as laser ablation are also often used [15]. Other examples of nanoparticle fabrication using physical methods include fabrication of nanostructured silver films by electron-beam evaporation [16], gas aggregation [17] and radio-frequency sputtering [18]. The advantages of certain physical methods over chemical methods include that there is no reagent contamination and that the monodispersity of fabricated NPs can be controlled very well [13].

One of the less commonly used physical methods for the fabrication of SERS active gold and silver nanoisland films is pulsed laser deposition (PLD) [19–25]. In PLD, the materials are deposited on a substrate through laser ablation from a target located opposite to the substrate. Deposition is typically performed in vacuum [20] or argon atmosphere [19] and by the change of parameters such as laser wavelength, pulse duration or laser fluence it is possible to modify the structure of the fabricated nanoislands. Until now, gold and silver nanoisland films have been fabricated by PLD using different lasers with different wavelengths and parameters [19–25]. The most commonly used lasers are KrF excimer lasers with a wavelength of 248 nm [20–24], however other wavelengths from UV (193 nm, 266 nm, 308 nm, 355 nm), through vis (527 nm, 532 nm [19]) to IR (1064 nm) have also been used. Even though several studies were reported on the PLD fabrication of plasmonic metal films, there are only a few recent studies discussing an influence of a wider range of deposition process parameters on the morphology and optical properties of the films [19]. There are no studies that correlate a high number of PLD process parameters to the SERS properties of fabricated plasmonic metal nanoislands films.

Herein, we report the results of studies on the influence of several parameters of the fabrication of silver nanoisland films (SNIFs) using PLD on their morphology, optical and SERS enhancement properties. We first describe the PLD fabrication process and the influence of the deposition process parameters on the morphology of the fabricated films determined by SEM and AFM measurements. Then, we present the results of measurements of the chemical composition of the fabricated SNIFs by using X-ray photoelectron spectroscopy (XPS) and their optical properties by using UV–vis spectroscopy. Finally, we describe the SERS performance of SNIFs in the measurements of *para*-mercaptoaniline (pMA) molecules.

## Results and Discussion

### Preparation of silver nanoisland films

We investigated ten samples with silver nanoisland films deposited under different conditions, designated “A” to “I”. The deposition processes were carried out using an ArF excimer laser (wavelength 193 nm) at the same pressure and laser repetition rate but at different temperatures of the resistively heated furnace on which the substrates were mounted (room temperature (RT), 190 ± 3 °C, 340 ± 3 °C), different numbers of laser pulses (1000, 2000, 4000, 8000, 16000) and with two laser fluence values (5.56 ± 0.37 J/cm^2^ or 2.52 ± 0.17 J/cm^2^) (Table 1).

**Table 1:** The parameters used during the deposition of the SNIFs by PLD (repetition rate 5 Hz; pressure 4.6 × 10^−5^ mbar).

sample	laser fluence [J/cm^2^]	number of laser pulses	temperature [°C]	calculated thickness of the layer [nm]

A	5.56 ± 0.37	1000	190 ± 3	9.0 ± 0.2
B	5.56 ± 0.37	2000	190 ± 3	17.9 ± 0.2
C	5.56 ± 0.37	4000	190 ± 3	35.8 ± 0.2
D	5.56 ± 0.37	8000	190 ± 3	71.6 ± 0.2
E	5.56 ± 0.37	16000	190 ± 3	143.2 ± 0.2
F	2.52 ± 0.17	1000	190 ± 3	4.7 ± 0.2
G	2.52 ± 0.17	2000	190 ± 3	9.3 ± 0.2
H	2.52 ± 0.17	1000	340 ± 3	4.7 ± 0.2
I	2.52 ± 0.17	2000	340 ± 3	9.3 ± 0.2

Temperature is the most important factor influencing the formation of SNIFs on the silicon substrates. An increase in the temperature of the substrate increases the kinetic energy of the silver atoms and results in a higher ordering of the structure, while the applied amount of laser pulses allows for the control of the dimensions of the obtained silver nanoislands.

In all experiments, the same laser repetition of 5 Hz was used. Increase or decrease of the repetition rate of the laser has a similar effect on the morphology as changing the laser pulse energy. The low laser repetition rate adopted in the experiment allowed us to examine the influence of other deposition conditions on the structure of SNIFs produced. The applied laser fluence and the number of laser pulses affect the structural parameters of the obtained SNIFs, such as the size and spacing of the silver nanoislands.

### Calculations of the growth rate of silver nanoisland films per laser pulse

**Figure 1:**
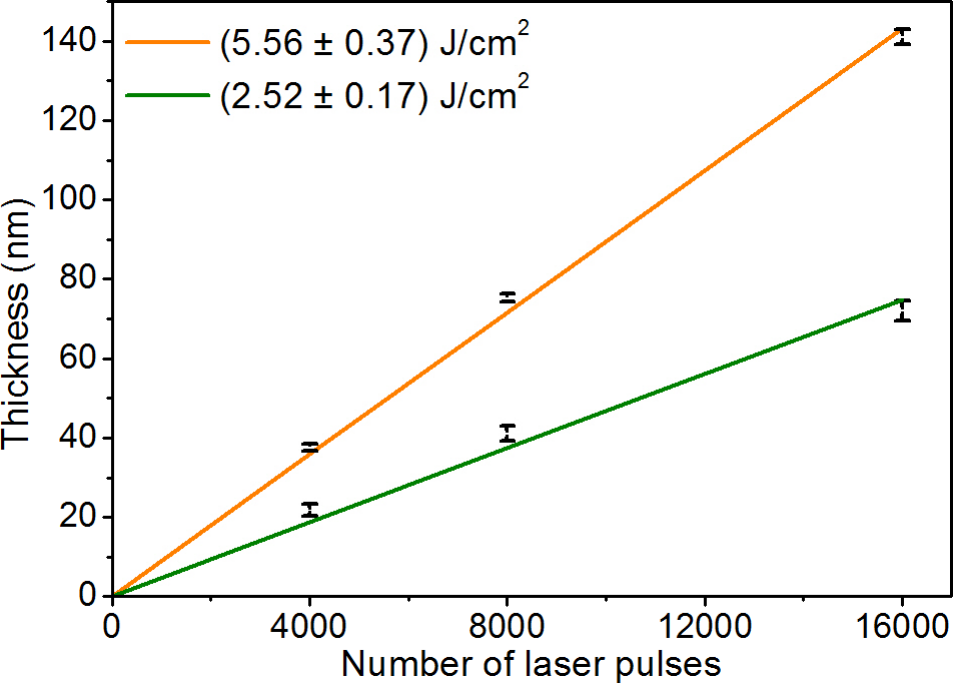
Thickness of the silver nanoisland films as a function of the number of applied laser pulses. Results obtained based on the AFM measurements of the reference samples prepared for laser fluences of 5.56 ± 0.37 J/cm^2^ and 2.52 ± 0.17 J/cm^2^.

The thickness of the silver nanoisland films as a function of the number of applied laser pulses is shown in Figure 1. The plot was made based on AFM measurements of reference Si samples with silver films deposited at room temperature for both fluences of laser radiation used (three samples for each fluence). The reference silver films had different thicknesses depending on the number of laser pulses used (4000, 8000, 16000 pulses). The dependence of layer thickness from the number of laser pulses was approximated by a linear function.

The growth rate of the silver films was calculated as the directional coefficient of the approximation function. In the case when the fluence of the laser radiation was 5.56 ± 0.37 J/cm^2^, the calculated layer growth rate was 9.0 ± 0.2nm per 1000 laser pulses. When the laser fluence was about half as low, 2.52 ± 0.17 J/cm^2^, the calculated growth rate was 4.7 ± 0.2 nm per 1000 laser pulses. The growth rates achieved permit calculation of the approximate thickness of the silver nanoisland films obtained (Table 1). Depending on the number of laser pulses used, the estimated thickness of the deposited layers ranges approximately from 4.7 ± 0.2 nm to 142 ± 0.2 nm (Table 1).

### Morphology and dimensions of silver nanoisland films

**Figure 2:**
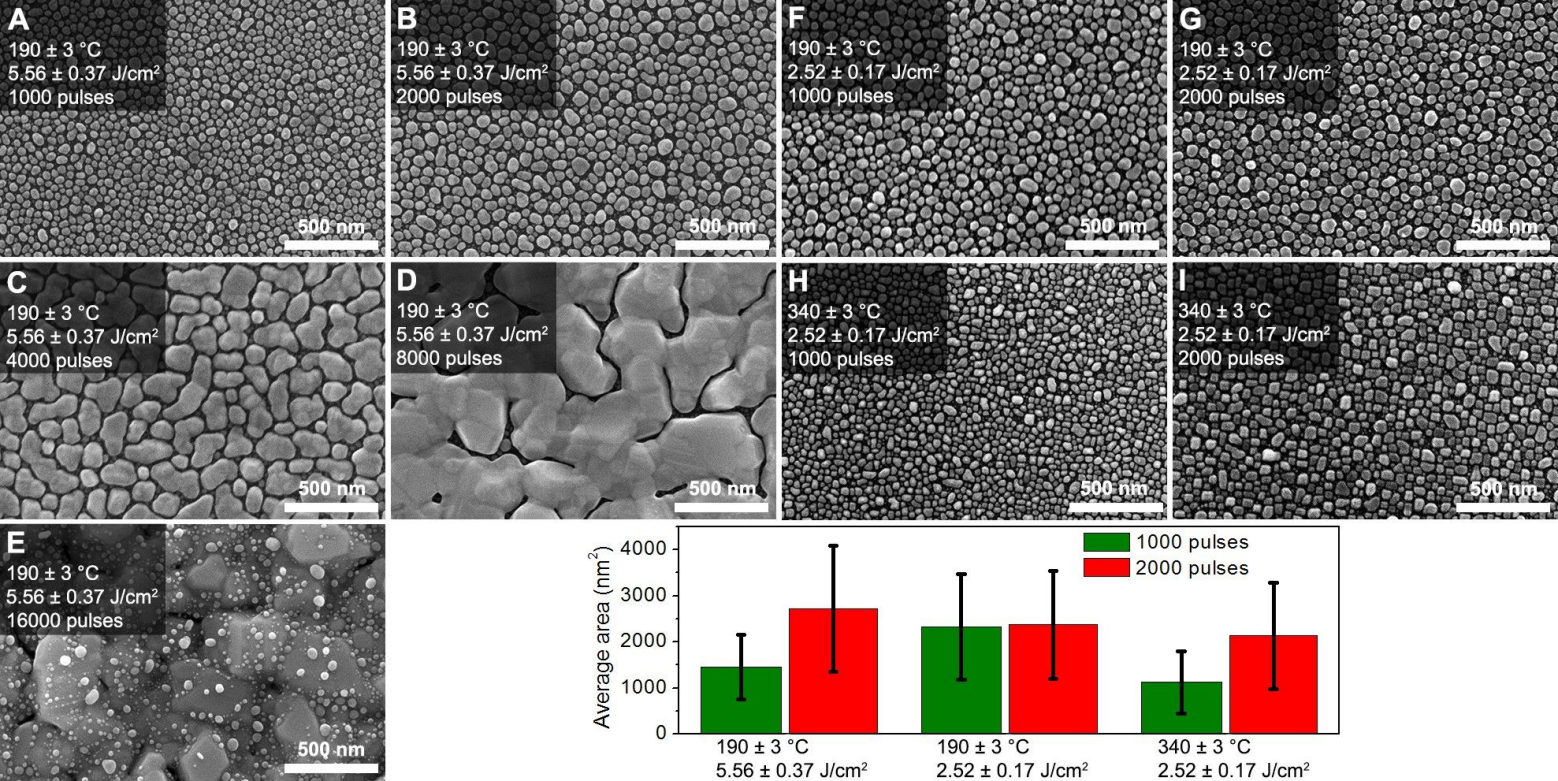
SEM images of the nanostructured silver films deposited on Si substrates by the PLD method using different process parameters. A bar graph showing the average surface of the silver nanoislands as a function of the deposition parameters for 1000 and 2000 laser pulses.

SEM images of the deposited silver nanoisland films are shown in Figure 2. In the case of silver deposition without heating the substrate (Figure S1 in Supporting Information File 1), the obtained silver film is continuous. In other cases, when the substrates are heated to 190 ± 3 °C and 340 ± 3 °C, silver nanoisland films are formed. This effect is related to the Volmer–Weber island growth model [26]. The thermal energy supplied to the silver atoms increases their kinetic energy and thus enables their diffusion and ordering on the surface of the substrate. The ordering of nanoislands, their shape and the homogeneity, strongly depends on the temperature. In the case when the temperature of the furnace is 190 ± 3 °C and the number of pulses does not exceed 2000 the shape of the silver nanoislands resembles the oval shape. At a temperature of 340 ± 3 °C, the islands that are formed have a clearly cubic shape (comparing Figure 2F to 2H and 2G to 2I) which is related to different dynamics of the layer growth process and translates into their higher crystallization.

The dimensions of the silver nanoislands are determined by the number of laser pulses used during deposition. Island dimensions increase from about 40 nm for sample A (Figure 2A) to 300 nm for sample C (Figure 2C). In the case of 4000 laser pulses for sample C, the coalescence of neighboring islands into larger structures with elongated shape (Figure 2C) is also visible. After 8000 laser pulses, the embedded silver islands are already that large that almost all of them are connected and a semicontinuous layer with an irregular structure is formed (Figure 2D). After 16000 laser pulses the nucleation and growth of silver nanoislands occurs on the semicontinuous silver layer formed earlier (Figure 2E). The change of laser fluence from 5.56 ± 0.37 J/cm^2^ to 2.52 ± 0.17 J/cm^2^ does not cause noticeable changes in the shape of the silver islands (comparing Figure 2A to Figure 2F and Figure 2B to Figure 2G), but it affects the size of the islands. The smaller fluence leads to smaller increase in layer thickness. Hence, the islands obtained have a slightly smaller size (comparing Figure 2B to Figure 2G).

The distributions of the surface areas of the fabricated silver nanoislands were determined based on the SEM images. For silver layers deposited with 1000 and 2000 laser pulses, the average surfaces area values of silver nanoislands were calculated. The analysis was not carried out for samples D and E because they can be characterized as continuous or semicontinuous films. The results are shown on Figure 2 together with error bars of one standard deviation from the average value of the silver nanoislands surface. The calculated average area of the silver nanoislands is 1448 ± 701 nm^2^, 2324 ± 1141 nm^2^ and 1126 ± 674 nm^2^ for samples A, F and H, respectively, prepared using 1000 laser pulses (green bars) and 2717 ± 1268 m^2^, 2375 ± 1169 nm^2^ and 2132 ± 1155 nm^2^ for the samples B, G and I, respectively, prepared using 2000 laser pulses (red bars). As can be seen from the presented data, both fluence and temperature of the resistively heated furnace have a large impact on the average surface area of the obtained silver nanoislands. After the reduction of the laser fluence by almost one half, which results in a proportional reduction of the growth rate of the layers, the average area of the silver islands is greater than one would expect. The lower fluence of laser radiation while maintaining the same temperature of the substrate (190 ± 3 °C) favors the growth of silver nanoislands with a larger area. In addition, the average area of silver islands for 1000 and 2000 laser pulses are also very similar. The process of silver nanoisland growth is even different when the furnace temperature rises to 340 ± 3 °C. Under these conditions, the average area of silver islands obtained for 2000 laser pulses is almost twice as high as for 1000 laser pulses. In addition, the average surface area of the obtained silver islands with the same number of laser pulses is smaller compared to the case of layer growth at a lower temperature.

**Figure 3:**
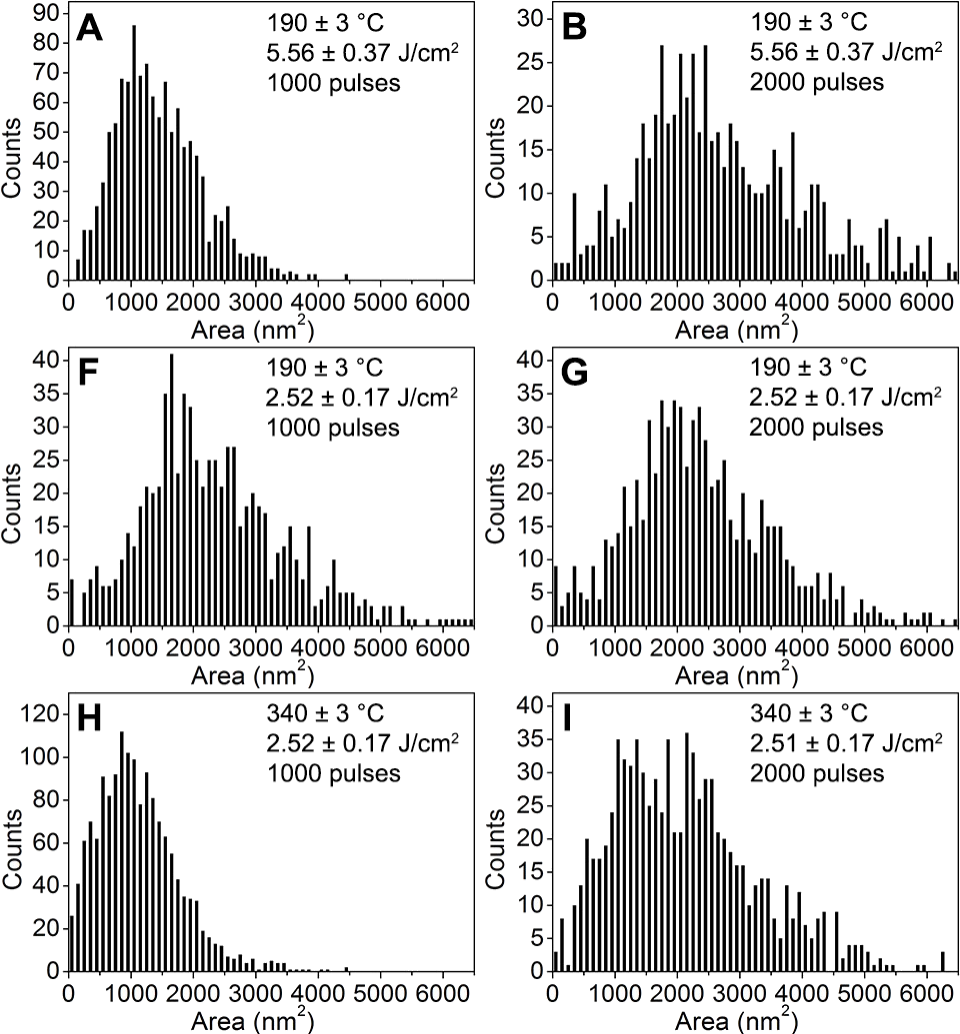
Histograms of the silver nanoisland surface areas made on the basis of SEM images.

A detailed analysis of surface distribution of silver nanoislands for samples A, B, F, G, H and I are shown in Figure 3 in the form of histograms. The calculated areas of silver nanoislands were grouped in compartments with a width of 100 nm^2^. The distribution of the surface of the silver islands varies from 0 to 6050 nm^2^. The dominant area of the silver nanoislands taken as the maximum of the histogram is about 1050, 2150, 1850, 1950, 850 and 1450 nm^2^ respectively for samples A, B, F, G, H and I. The narrowest distributions of the surface area of the silver nanoislands were obtained for samples A and H with SNIFs deposited using the smallest number of laser pulses. Comparing the histogram obtained for sample H with samples F or A, it is also apparent that the deposition of silver layers on substrates with a higher temperature leads to the formation of more homogeneous and smaller silver nanoislands.

We also assume that the deposition conditions of the SNIFs determine the distances between the Ag nanoislands and the formation of so called hot spots. These areas are created in places where the distances between neighboring particles are equal to 2–5 nm. The results of the SERS measurement discussed in further sections of this article suggest that the distances between the silver nanoislands are increasing as the temperature of the substrate increases. This conclusion is consistent with the observed reduction of the enhancement factor (EF) achieved for the Raman signal when the substrate temperature rises.

### Chemical composition of the silver nanoisland films

**Figure 4:**
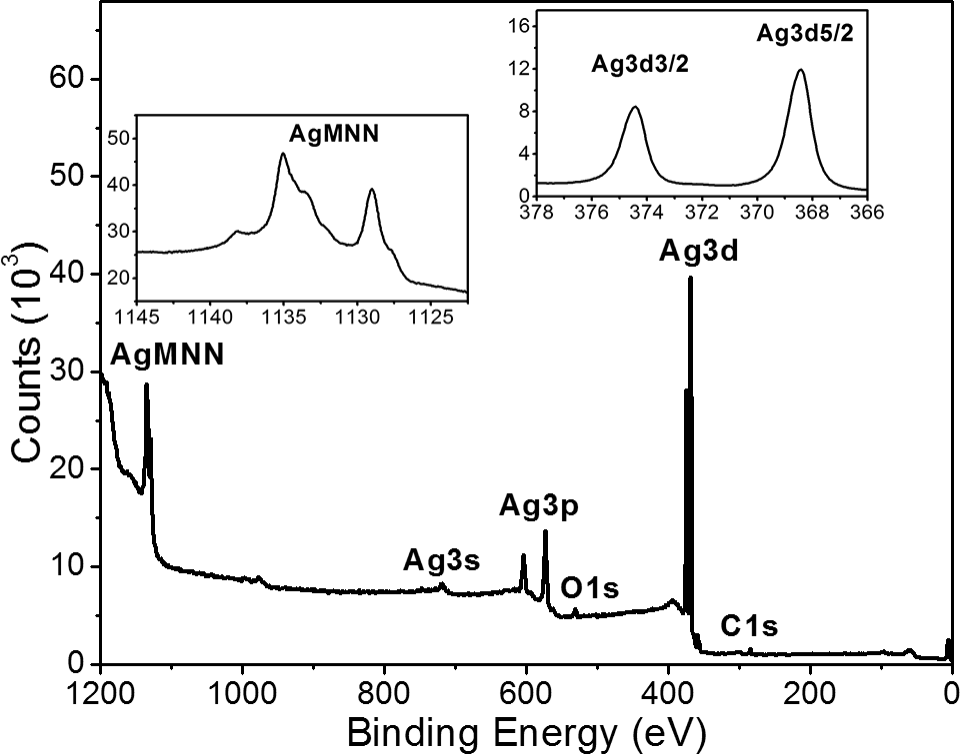
XPS spectra of silver nanoisland films deposited on the Si substrate by laser ablation (PLD) registered in a wide range of binding energy, and Ag 3d and Ag-MNN Auger band registered in a narrow range of energy (insert).

The chemical composition of the PLD-deposited silver nanoisland films was investigated by using XPS spectroscopy. The results of XPS measurements are shown in Figure 4. The XPS spectrum registered over a wide range of binding energy indicates that in addition to silver there are small amounts of carbon and oxygen impurities. These impurities may be located on the silicon substrates used. The position, shape and half-width of peaks registered for the Ag 3d band are typical for silver in metallic form: Ag 3d_3/2_ – 374.16 eV, FWHM 0.97 eV; Ag 3d_5/2_ – 368.16 eV, FWHM 0.96 eV. This is also confirmed by the spectra of the Auger band, which is typical for metallic silver [27]. The metallic form of silver has also been confirmed by comparing the recorded spectra of the sample with the spectra recorded for Ag foil with 99.95% purity. XPS studies therefore confirm that silver deposited by the PLD method does not oxidize during deposition under vacuum conditions.

### Optical properties of fabricated Ag nanoisland films

**Figure 5:**
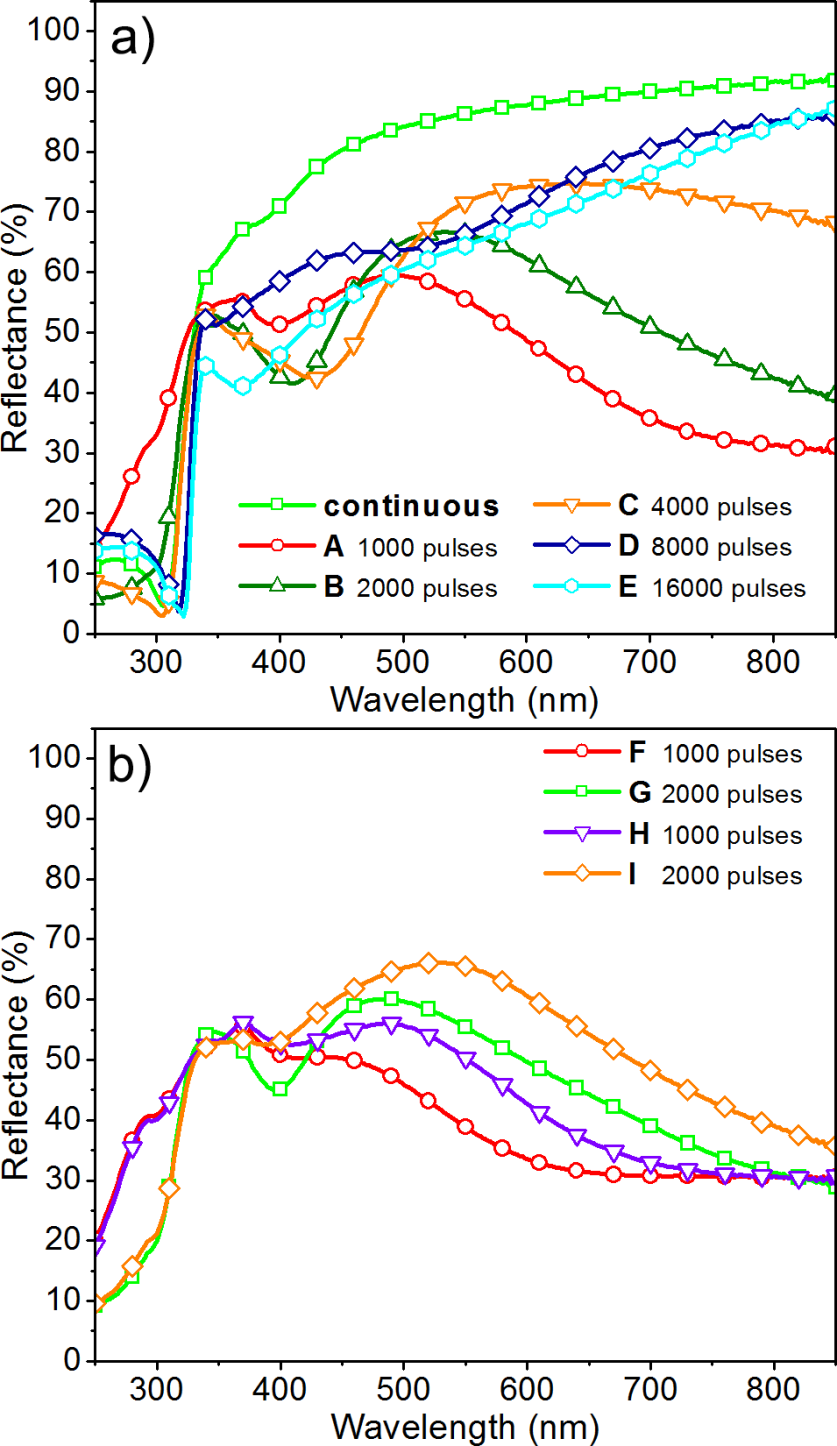
Reflectance spectra of fabricated Ag nanoisland films: a) for samples with the layers deposited at a laser fluence of 5.56 ± 0.37J/cm^2^, b) for samples with the layers deposited at a laser fluence of 2.52 ± 0.37J/cm^2^.

The UV–vis spectra of fabricated silver nanoisland films are shown in Figure 5. In the case of a continuous layer of silver (4000 pulses, RT), the monotonically increase of the reflectance is visible as the wavelength increases. Samples D and E, with the thickest layers of Ag, show similar shapes of the UV–vis spectra. These samples, however, have a lower reflectance in the range of 350 to 850 nm and local minima at around 540 nm and 370 nm,respectively.

The samples with the smallest dimensions of silver nanoislands (samples A, B, F, G, H, and I) have completely different shapes of spectra. These samples have a much lower reflectance in the range of 350 to 850 nm with one characteristic minimum between 400 and 430 nm, which corresponds to plasmon resonance of Ag NPs. The small shift of the plasmon resonance peak may be related to rather small variations of the size of particles between samples obtained using various deposition process parameters. Then reflectance increases and reaches a maximum in the range of 450 to 520 nm and decreases again towards the infrared region. The reflectance above 500 nm depends on the number of laser pulses and the temperature of the substrate and increases with the increase in the number of pulses and the temperature of the substrates. This is because of the growing size of the silver nanoislands and the strong coupling between them. The strange behavior of samples B, G and I at lower wavelengths (270–280 nm) may be associated with the optical properties of the silicon substrate. Because silicon has the maximum of reflectance in this area, its effect is visible in the spectra of the thinnest silver films.

### SERS activity of fabricated silver nanoisland films

**Figure 6:**
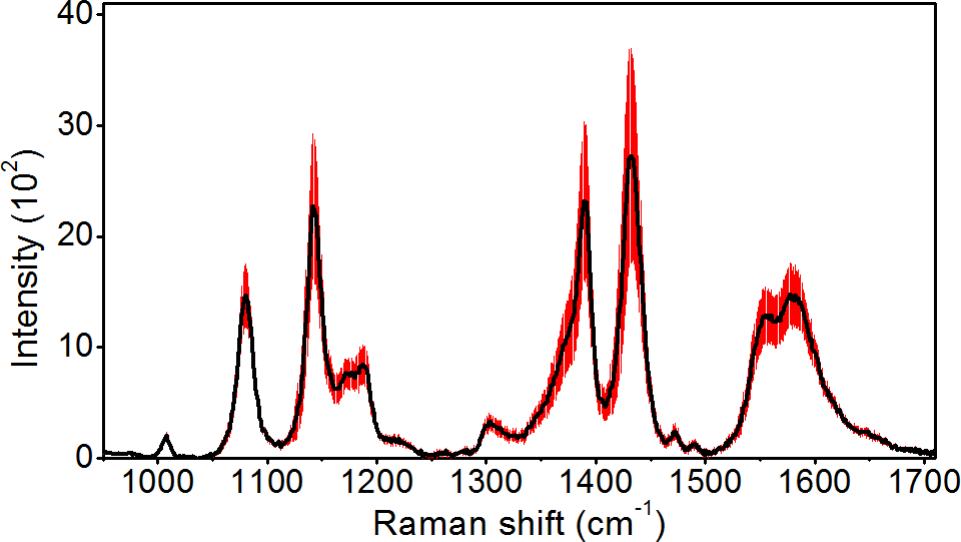
SERS spectrum recorded for sample B (black line – the average SERS spectrum; red area – the standard deviation of the signal.

All fabricated Ag films were tested to determine their suitability for SERS measurements using pMA as a test analyte. An example of averaged SERS spectra for sample B is presented in Figure 6. The black line represents the average spectrum and the red shade represents the standard deviation around the average spectrum. This spectrum was obtained as a result of averaging the SERS spectra from 351 measurement points.

For registered maps composed of 351 measurement points a statistical analysis was made determining the average intensity of the peak at 1080 cm^−1^ and the standard deviation of the peak intensity. Then based on the standard deviation of the 1080 cm^−1^ peak intensity, for each measurement, the relative intensity deviation was calculated. The evaluation of the average intensity of the 1080 cm^−1^ peak and its relative intensity deviation allows us to determine which samples have the highest EF of the Raman signal and the highest EF repeatability. Results of the statistical analysis of the SERS spectra obtained are presented in Table 2.

**Table 2:** Parameters of nanostructured silver layers determined based on SERS measurements.

sample	excitation wavelength 532 nm (measurement parameters: 75 µW, 1 s)			Excitation wavelength 633 nm (measurement parameters: 27 µW, 5 s)		
	average intensity of peak 1080 cm^−1^	relative intensity dev. (%)	EF (×10^3^)	average intensity of peak 1080 cm^−1^	relative intensity dev. (%)	EF (×10^3^)

A	996	11	276.3	2141	21	25.3
B	1455	19	403.7	3098	17	36.6
C	1886	18	523.4	2637	52	31.1
D	2086	68	578.8	1375	67	16.2
E	1446	29	401.4	1840	51	21.7
F	843	12	233.8	1427	20	16.9
G	2440	17	677.2	4673	20	55.2
H	474	6	131.5	611	6	7.2
I	757	7	210.1	1000	6	11.8

As we can see from Table 2, the average peak intensity and relative intensity deviation depend on the size of the silver nanoislands obtained. In the case of samples deposited with a laser fluence of 5.56 ± 0.37 J/cm^2^ and a substrate temperature of 190 ± 3 °C, with an excitation wavelength of 532 nm the average intensity of the peak increases from 996 for sample A to 2086 for sample D and then slightly decreases to 1446 for sample E. When we compare this data with the SEM images (Figure 2), an increase in the average peak intensity is correlated with the increase in the size of the silver nanostructures, until the connection of silver nanoislands into one structure occurs. With the increase in the size of the silver nanoislands, the relative intensity deviation of the peak increases also. As a result, sample A has the smallest relative intensity deviation value of 11%, whereas for sample D this parameter reaches the highest value of 68%. In the case of sample E having a smaller average intensity of the peak at 1080 cm^−1^, the relative intensity deviation also decreases to 29%. This means that the layers with the smallest size of the silver nanoislands have the highest EF reproducibility, but at the same time they have a smaller EF of the Raman signal. A similar trend in changes in the intensity of the peak at 1080 cm^−1^ is visible for 633 nm excitation. In this case, however, sample B has the highest intensity of the peak at 1080 cm^−1^ and then it decreases when the dimensions of the silver nanoislands increase. Also, the relative intensity deviation values obtained for 633 nm excitation are very similar to the values obtained for 532 nm excitation.

In the case of a lower fluence of the laser radiation 2.52 ± 0.17 J/cm^2^ and the two temperatures of the substrate 190 ± 3 °C and 340 ± 3 °C, the situation is more complicated. For both excitation wavelengths, the highest 1080 cm^−1^ peak intensity was obtained for sample G (2440 and 4673 for excitation wavelengths of 533 and 633 nm, respectively). These values are also the largest in the group of all examined samples. It is important to note that reducing the rate of deposition of silver layers also leads to a reduction of the relative intensity deviation. The relative intensity deviation ranges, for both excitation wavelengths, from 12% to 20%, which means that the layers obtained are characterized by a greater uniformity of the enhancement factor of the Raman signal. In turn, the use of a substrate temperature of 340 ± 3 °C, resulting in a high crystallization of silver nanoislands, leads to a very large decrease in the intensity of the recorded peak for both excitation wavelengths. In this case, however, the obtained relative intensity deviation is the lowest of all samples and ranges from 6 to 7%.

The usefulness of the prepared SNIFs as SERS substrates depends on the enhancement factor achieved for the Raman signal. Several ways to calculate the EF have been reported in the literature [23,28–29]. In the simplest case, the EF is determined as the intensity ratio of the selected peak for the tested compound in the form of a monolayer on the SERS substrates and in the bulk form [23]. This method, however, is very inaccurate because of the different number of measured molecules of the tested substance contained in a monolayer and in the volume of the material. To obtain better results, the EF was calculated based on the Raman spectrum registered for a pMA monolayer adsorbed on the surface of a platinum foil with 99.998% purity. According to literature reports, a pMA monolayer can be made on the surface of platinum, as well as on the surface of silver and gold, a pMA monolayer can be made [30–31]. EF values were calculated according to the procedure described in Supporting Information File 1. In our EF calculations we have assumed that the intensity of the Raman pMA signal on Pt increased due to the chemical factor of 10^2^ and corrected the obtained EF values by this number [32–33].

The values of the EF of the Raman signal for SNIFs were determined based on the intensity of the peak located at 1080 cm^−1^, which in the pMA molecule corresponds to vibrations of the C–S bond [34]. The calculated average values of EF of the Raman signal are presented in Table 2.

As mentioned above, the average EF of the Raman signal depends on the wavelength of the excitation radiation. In the case of 633 nm excitation, the calculated average EF changes from 7.2 × 10^3^ to 55.2 × 10^3^ and sample G exhibits the highest EF of 55.2 × 10^3^. The average EF obtained for 532 nm excitation is 11-times to 36-times higher than that of the 633 nm excitation and takes values from 131.5 × 10^3^ to 677.2 × 10^3^. For this excitation wavelength, sample G also shows the largest average EF.

Samples C and D with the largest Ag nanoislands or semicontinuous Ag layers also show high EF values in the range of 532.4 × 10^3^ to 578.8 × 10^3^. Considering also the relative intensity deviation it can be concluded that deposited SNIFs differ from each other in the number of active SERS sites and their EF. The average SERS activity of a sample with a large number of SERS active sites but with low EF can be lower than the one of a sample with few active sites but with high EF. This may be the reason for the increase of EF and its relative intensity deviation in the case of samples A to F. With the increase in the number of laser pulses silver nanoislands get larger and their mutual distance decreases, which leads to the formation of gaps where the EF can reach higher values. If the deposition goes on, Ag islands start to coalesce and the number of gaps decreases. However, the smaller dimensions of the gaps can lead to higher EF values. At the same time, the SERS active sites are characterized by an increasing spread of EF. Thus the highest EF and relative intensity deviation of sample D can be due to the formation of highly SERS active sites (in the gaps) but with a non-homogenous spatial distribution. The SERS activity of samples F, G and H, I behaves similar to that of samples A–E as the number of laser pulses increases. However, the use of a lower laser fluence during the deposition changes the kinetics of Ag nanoisland growth, which gives a higher average EF value while maintaining the relative intensity deviation at 17%. In contrast, an increase in the temperature of the furnace to the 340 ± 3 °C (samples H and I) leads to a reduction of EF to less than half the value of the layers deposited at the same number of laser pulses but at a lower substrate temperature. A similar dependence of the EF change on the silver layer deposition conditions was observed for the 633 nm excitation. In this case, however, the EF of samples C and D is lower than the values expected from the results obtained with excitation at 532 nm. In summary, it should be noted that the EF calculation confirms the possibility of using silver nanoisland films deposited by the PLD method on silicon wafers as a SERS substrate.

**Figure 7:**
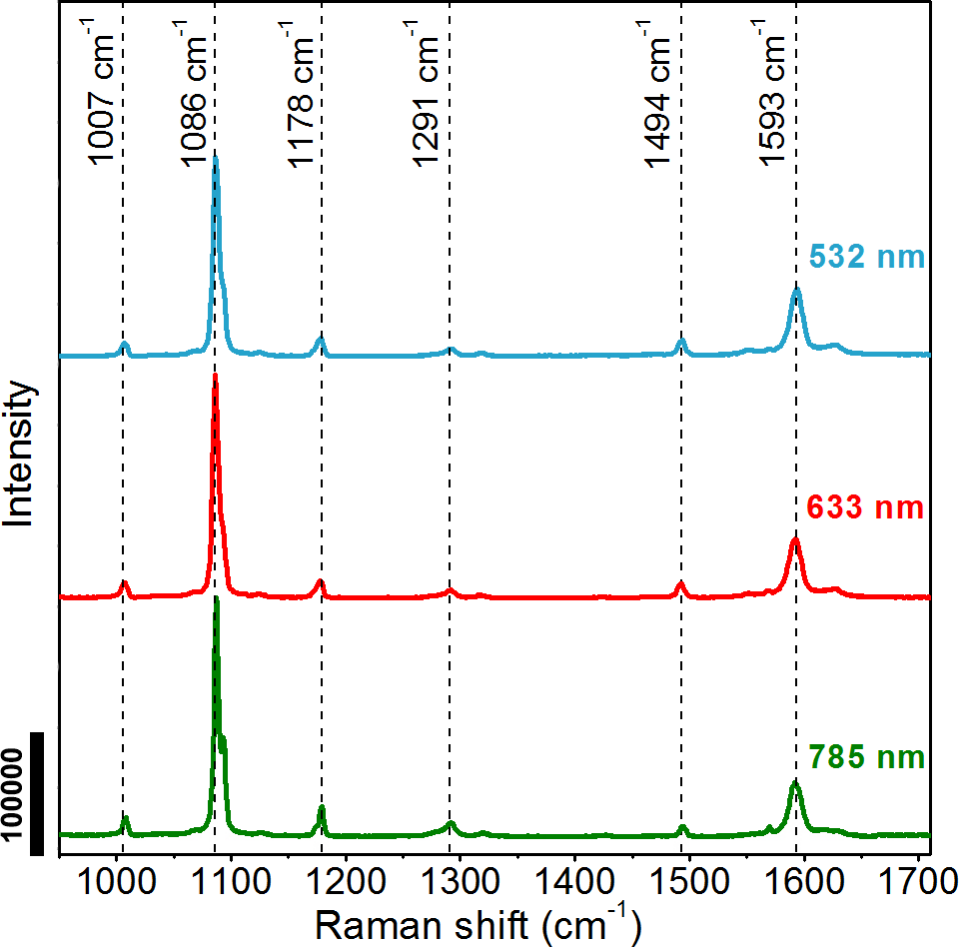
Comparison of the Raman spectra of the bulk pMA recorded using three excitation wavelengths of 532, 633 and 785 nm.

**Figure 8:**
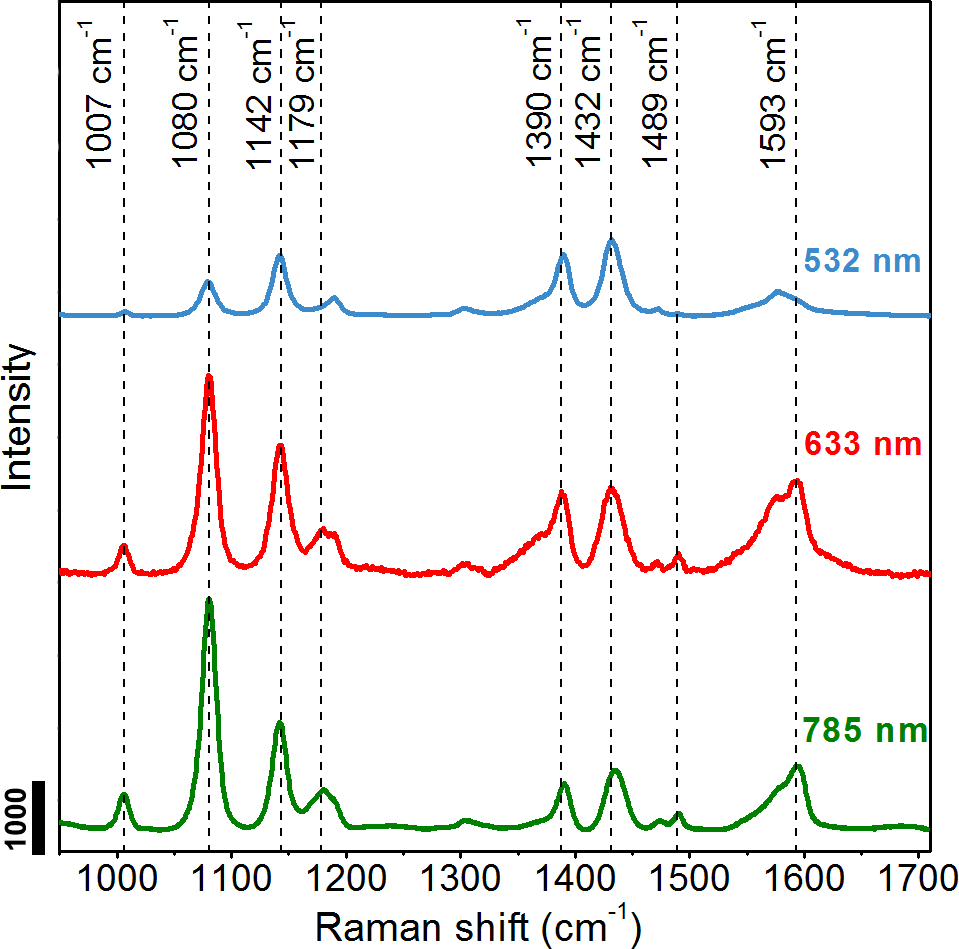
Comparison of the SERS spectra of the pMA monolayer formed on the silver nanoislands film (sample C) recorded using three excitation wavelengths of 532, 633 and 785 nm.

The SERS spectra of materials adsorbed on the surface of SERS substrates are usually different from Raman spectra of the bulk materials. In our research the SERS spectra of pMA adsorbed on the surface of the SNIFs have a different shape, number of peaks, peak position and intensity than Raman spectra of bulk pMA. A detailed analysis of this phenomenon was made based on Raman and SERS spectra recorded for sample C using three excitation wavelengths of 532, 633 and 785 nm (Figure 7 and Figure 8).

In the Raman spectrum of bulk pMA, recorded in a range of 950 to 1700 cm^−1^, there are two main peaks with the highest intensities at 1086 and 1593 cm^−1^ and four peaks with low intensities (at 1007, 1178, 1291 and 1494 cm^−1^) (Figure 7). The recorded Raman spectra are very similar regardless of the excitation wavelength. As shown in Table 3, the peak intensity ratios vary in a small range when the excitation wavelength is changed. For example, the intensity ratio for peaks 1086 and 1007 cm^−1^ is 9.66, 9.79 and 7.17 for 532, 633 and 785 nm excitation respectively. The spread of the intensity ratio of the peak at 1086 cm^−1^ relative to the other peaks is also very similar for all excitation wavelengths.

**Table 3:** Comparison of the intensity ratio of selected peaks in the Raman spectrum of bulk pMA and SERS spectrum of pMA monolayer on the surface of sample C.

pMA monolayer on the surface of sample C			
intensity ratio	excitation wavelength 532 nm	excitation wavelength 633 nm	excitation wavelength 785 nm

*I*_1080_/*I*_1007_	8.1	6.6	6.4
*I*_1080_/*I*_1142_	0.6	1.6	2.1
*I*_1080_/*I*_1179_	1.9	4.3	5.8
*I*_1080_/*I*_1432_	0.4	2.3	5.1
*I*_1080_/*I*_1489_	15.9	9.1	15.0
*I*_1080_/*I*_1593_	1.4	2.1	3.6

bulk pMA			
intensity ratio	excitation wavelength 532 nm	excitation wavelength 633 nm	excitation wavelength 785 nm

*I*_1086_/*I*_1007_	9.7	9.8	7.2
*I*_1086_/*I*_1178_	7.9	8.9	5.2
*I*_1086_/*I*_1494_	8.4	10.2	9.9
*I*_1086_/*I*_1593_	2.8	3.4	3.4

In the case of monolayers of pMA in sample C, large changes in the shape of the recorded SERS spectra were observed (Figure 8). First, in a range of 950 to 1700 cm^−1^, the number of peaks increased. In Figure 8 three additional high-intensity peaks appear at 1142, 1390 and 1432 cm^−1^. All observed peaks also slightly change their position relative to bulk pMA. Most striking, however, is the strong increase in the half-width of the registered peaks and their change of intensity relative to the 1080 cm^−1^ peak. The intensity of the peaks and the ratio of their intensities depend more on the wavelength of the excitation radiation (Table 3). The biggest changes are visible in the case of 532 nm excitation in which the main peak located at 1080 cm^−1^ has a lower intensity than the remaining peaks in the spectrum. This relationship is inverse compared to the other excitation wavelengths. The spectra obtained at 633 nm and 785 nm excitation, however, are very similar to each other [35]. A similar effect was reported by Jian Ye and co-authors for 4-ATP (4-aminothiophenol) and 4-MOTP (4-methoxy-thiophenol) [36]. The authors suggested that because of the change in the wavelength of the excitation laser and the use of the shorter wavelengths an extra non-electromagnetic enhancement effect occurs. This effect is visible during excitation with 633 nm, but the enhancement becomes even more pronounced when the excitation wavelength shifts to 532 nm. As it is known, the total enhancement of the Raman scattering signal in the SERS phenomenon depends on the EM (electromagnetic) and CT (charge transfer) effect. In the CT mechanism the charge transfer between the molecules of the analyte and metallic nanostructures is excited, which leads to a resonant increase in the total EF. When the laser energy matches the energy gap between the HOMO and LUMO of molecules, a direct resonant Raman scattering can be excited in the general CT mechanism. On the other hand a “resonance Raman-like” process can appear as result of an indirect coupling by CT through the metal [37–38]. The authors of the publication [36] attributed the effect of changing the shape of the band in the SERS spectra for the CT effect, the contribution of which to the total enhancement can be different for different Raman bands and depends also on the excitation wavelength as well as the structure of the molecule. However, other publications also suggest the formation of new intense peaks and a shape change of the bands is a result of photo-induced chemical transformation or plasmon-assisted (or ‘‘hot electrons’’) catalytic reaction of molecules [39–40].

## Conclusion

In this work, we have shown that pulsed laser deposition (PLD) with simultaneous heating of the substrate permits the controlled fabrication of silver nanoisland films with good SERS properties. Dimensions and shapes of silver nanoislands can be controlled by varying the temperature of the substrate and the fluence of the laser radiation, while the thickness of the deposited layers is determined by the number of laser pulses. This method allows for the production of silver nanoislands with good homogeneity in shape and size in different areas of the films, with very sharp edges on the borders of nanoislands and small distances between them. The results of the XPS measurements confirm that the silver PLD-deposited occurs only in metallic form. Acquisition of Raman spectra of pMA molecules adsorbed on the fabricated silver nanoisland films showed that these nanostructures strongly amplify the Raman signal from adsorbed molecules. The best SERS performance (the highest enhancement factor) was observed for SNIFs deposited at a temperature of 190 ± 3 °C, laser fluence 2.52 ± 0.17 J/cm^2^ and 2000 laser pulses. The average EF of the Raman signal for the substrate prepared under these conditions was 677.2 × 10^3^ for 532 nm excitation and 55.2 × 10^3^ for 633 nm excitation. The highest homogeneity of SNIFs was obtained by using lower laser fluences, smaller numbers of laser pulses and a substrate temperature of 340 ± 3 °C.

## Experimental

### Deposition of nanostructured layers of silver

Silver nanoisland films were fabricated on an n-type doped silicon substrate with orientation <100> and dimension 10.0 × 10.0 × 0.5 mm. The SNIFs were prepared by using pulse laser deposition (PLD) with an ArF excimer laser (LPX 305i, Lambda Physik Company). The laser used in the experiment is characterized by the following parameters: λ = 193 nm, *E* = 700 mJ, τ ≈ 15–20 ns.

Deposition was performed in a vacuum chamber at a pressure of around 4.6 ± 0.65 × 10^−5^ mbar and with different temperatures of the resistively heated furnace in which the substrates were mounted (room temperature (RT), 190 ± 3 °C, 340 ± 3 °C) (Table 1).

A rotating silver target with a purity of 99.95% was used. The laser beam was focused on the target at an incident angle of 45° and the distance between the target and the substrate was constant and equal to 65.0 ± 0.5 mm. The measured area of the laser spot on the surface of the target was 6.12 ± 0.17 mm^2^. Laser fluence on the target surface, calculated based on the area of the laser focus and energy of the laser pulse, was 5.56 ± 0.37 J/cm^2^ or 2.52 ± 0.17 J/cm^2^ (Table 1). Change in fluence of the laser pulse leads to a change in the mass of silver deposited per laser pulse. This, in turn, affects the rate of growth of the layer and the size and shape of the formed silver nanoislands.

### Characterization of nanostructured silver layers

The morphology of the deposited silver layers was visualized using a scanning electron microscope (SEM, Quanta 3D FEG, FEI Company). SEM images were also used for the surface analysis of the obtained silver islands. The analysis was carried out in the Gwyddion software dedicated to the processing and visualization of scanning probe microscopy images.

The thickness of the deposited reference silver layers was measured with an atomic force microscope (AFM, NT-MDT Company) in non-contact mode. To perform AFM measurements, the silver layers were removed in random places on the sample by scratching its surface with a sharp needle. A very sharp edge for height (layer thickness) measurements was obtained in this way due to the low adhesion of the silver films to the Si substrate. AFM measurements were carried out in three different areas on the surface of each sample. Then for each sample ten AFM cross sections from different scanning areas were made and averaged. As a result, the average layer thickness and the standard deviation of thickness were determined for each sample. The determined thicknesses of reference silver films were then used to prepare the graph shown in Figure 1.

To determine the chemical composition of the deposited silver layers X-ray photoelectron spectroscopy (XPS) was used (XPS spectrometer, Prevac Company). The measurements were made using an X-ray source equipped with an Al anode emitting X-ray radiation with photon energy of 1486.6 eV. The analysis of registered XPS spectra was performed in the CasaXPS software.

UV–vis reflectance spectra were measured at room temperature using a Lambda 650 UV–vis spectrophotometer (Perkin Elmer), equipped with a 150 mm integrating sphere, in the 250–900 nm spectral range with increment of 2 nm. Due to the lack of transmission through the applied substrates, measurements were carried out on the samples placed behind the integrating sphere. In this configuration the reflectance spectrum was recorded for each sample.

### Raman and SERS measurements

Both Raman and SERS measurements were carried out using Renishaw InVia Raman microscope equipped with an EMCCD (1600 × 200 pixels) detector. The Raman signal was acquired using laser radiation with a wavelength of 532, 633 and/or 785 nm. The laser excitation power on the sample depended on the type of measurement and the laser wavelength used. The laser beam was directed to the sample through a 100× (NA = 0.85) objective lens. For the lens used, the diameter of the measuring point from which the Raman signal is recorded was approximately 1 μm. The wavelength of the instrument was calibrated using an internal silicon wafer, and the spectrum was centered at 520.5 cm^−1^.

Raman spectra of bulk pMA were measured first to compare them with the SERS spectra obtained on SNIFs substrates. For pMA bulk, measurements were made on excitation lengths of 532, 633 and 785 nm. The laser beam power measured on the surface of the pMA bulk was 151 ± 5 µW, 269 ± 11 µW and 464 ± 32 µW for the excitation with 532, 633 and 785 nm, respectively. The acquisition time and the number of acquisitions for all excitation wavelengths were set to 10 s and 5 s, respectively.

For SERS measurements, the pMA monolayers were deposited on the fabricated silver nanoislands. For this purpose, samples with deposited SNIFs were placed in Petri dishes and 2 mL of 0.01 M pMA solution in ethanol was added to the dish. The samples were left in solution for about 60 min. After removing the samples from the solution, they were rinsed twice with pure ethanol and then allowed to dry.

SERS spectra of all samples A–I were recorded using two lasers with wavelengths of 532 and 633 nm. Additionally, for sample C SERS spectra were also recorded at 785 nm excitation. The laser beam power measured on the surface of the samples was 75 ± 3 µW for 532 nm, 27 ± 1 µW for 633 nm excitation, and 464 ± 32 µW for 785 nm excitation (sample C). The acquisition times for a single point were 1 s, 5 s and 0.5 s for 532 nm, 633 nm, and 785 nm excitation wavelengths, respectively. The measurement parameters were adjusted to different nominal output power values of the lasers to obtain a good signal-to-noise ratio for a single measuring point. SERS measurements were made for maps consisting of 351 points per sample. Based on them the average spectrum was obtained and the standard deviation of the signal was determined, which is an indicator of the homogeneity of the deposited layers and Raman signal amplification obtained. Statistical analysis of the intensity of the recorded spectra and their standard deviation were made for the 1080 cm^−1^ peak. The average intensity of the peak calculated based on 351 measurement points and the standard deviation of the intensity allow us to determine which samples have the highest Raman signal amplification and highest homogeneity of the silver nanoisland films. To facilitate the evaluation of the reproducibility of Raman amplification, a calculation of the relative intensity deviation was also made. These parameters were counted as a ratio of standard deviation intensity and average intensity of the 1080 cm^−1^ peak.

To determine the EF of the Raman signal, the Raman measurements of the pMA adsorbed on the surface of the platinum film were also done. Compared to SERS substrates, obtaining a spectrum with the appropriate signal-to-noise ratio required increasing the laser power and extended measurement time. For the 532 nm excitation, a laser power of 755 ± 26 μW was used and the measurement time for one point was 100 s. In contrast, for the 633 nm excitation, a laser power of ca. 54 ± 2 μW was used and the measurement time for one point was 100 s. In both cases, measurements were taken at several points and the average spectrum was taken as a result. The EF values for the SERS substrate were calculated taking into account coefficients of proportionality arising from different laser power and different measurement times. All spectra were background-corrected before EF calculation.

## Supporting Information

File 1 SEM image of continuous Ag film, description of procedure of the determination of the enhancement factor (EF) and Raman spectrum of pMA on platinum foil.
