# Supplementary material for: Fabrication of silver nanoisland films by pulsed laser deposition for surface-enhanced Raman spectroscopy
Source: Beilstein J Nanotechnol. 2019 Apr 16;10:882–93. doi: 10.3762/bjnano.10.89 (PMC6541365; doi:10.3762/bjnano.10.89)
Supplement: File 1 — SEM image of continuous Ag film, description of procedure of the determination of the enhancement factor (EF) and Raman spectrum of pMA on platinum foil. [file Beilstein_J_Nanotechnol-10-882-s001.pdf]

BEILSTEIN JOURNAL OF NANOTECHNOLOGY

Supporting Information

for

Fabrication of silver nanoisland films by pulsed laser deposition for surface-enhanced Raman spectroscopy

Bogusław Budner, Mariusz Kuźma, Barbara Nasiłowska, Bartosz Bartosewicz, Malwina Liszewska and Bartłomiej J. Jankiewicz

Beilstein J. Nanotechnol. **2019,** *10,* 882–893. doi:10.3762/bjnano.10.89

SEM image of continuous Ag film, description of procedure of the determination of the enhancement factor (EF) and Raman spectrum of pMA on platinum foil

License and Terms: This is a supporting information file under the terms of the Creative Commons Attribution License (http://creativecommons.org/licenses/by/4.0). Please note that the reuse, redistribution and reproduction in particular requires that the authors and source are credited.
The license is subject to the *Beilstein Journal of Nanotechnology* terms and conditions: (https://www.beilstein-journals.org/bjnano)

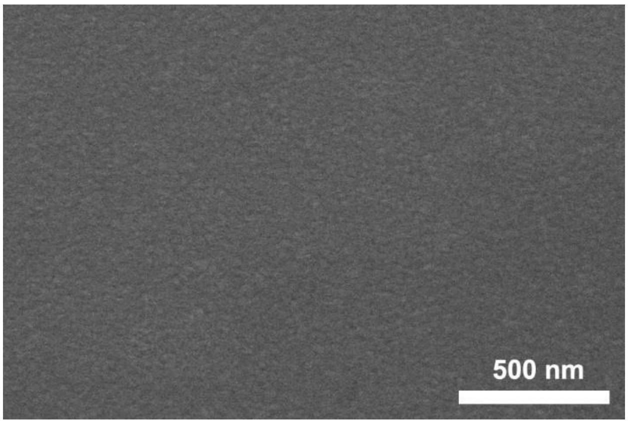

Figure S1: SEM image of a continuous silver layer deposited using 4000 laser pulses at room temperature.

Determination of the enhancement factor (EF)

The pMA monolayer on the platinum surface was prepared in the same manner as on the SNIFs deposited by the PLD method. Raman spectra for the pMA monolayer on the platinum foil recorded for excitation wavelengths of 532 and 633 nm are shown in Figure S2. The spectra based on which EF of the Raman signal were calculated come from the averaging of several spectra recorded on the surface of the platinum foil. Compared to bulk pMA, recorded spectra have a different shape, position and number of peaks. The calculation involved the intensity of the 1077 cm^{-1} peak corresponding to the 1086 cm^{-1} peak for bulk pMA.

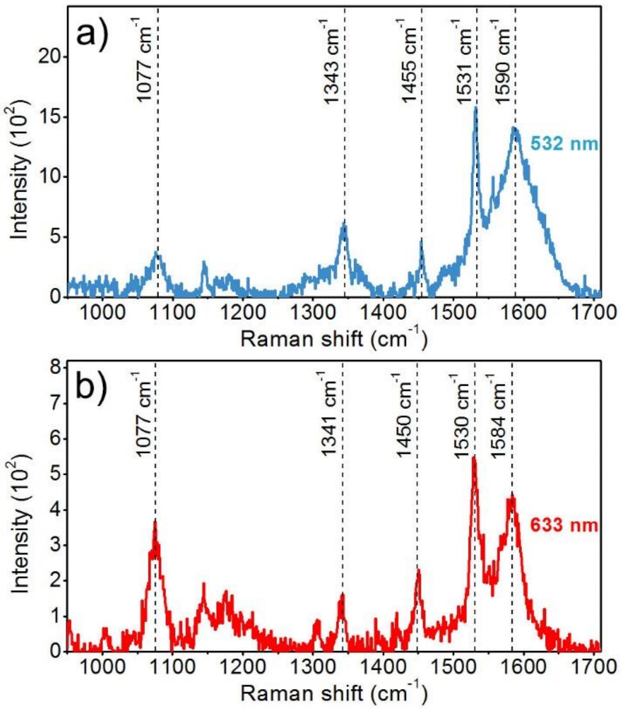

Figure S2: Raman spectra of the pMA monolayer formed on the platinum foil, recorded using two excitation wavelengths: a) 532 nm, b) 633 nm.

The correct calculation of the average EF of the Raman signal requires the following formula:

$$EF = \frac{I_{SERS}/N_{SERS}}{I_{Pt}/N_{Pt}}$$

where: $\boldsymbol{I_{SERS}}$ - the intensity of 1080 cm^{-1} peak in the SERS spectrum;

$\boldsymbol{N_{SERS}}$ - the average number of adsorbed molecules on the SERS substrate from which the signal was collected;

$\boldsymbol{I_{Pt}}$ - the intensity of 1077 cm^{-1} peak in the Raman spectrum of pMA monolayer on the platinum foil;

$\boldsymbol{N_{Pt}}$ - the average number of adsorbed molecules on the platinum foil from which the signal was collected.

Knowing that p-mercaptoaniline forms a monolayer on the silver surface and on the surface of the platinum foil [1], it was assumed that the number of pMA molecules on

the tested surface in both cases is the same. With this assumption, the N_{SERS} and N_{Pt} values are equal and the formula for the EF takes this simpler form:

$$EF = \frac{I_{SERS}}{I_{Pt}}$$

References

1. Kudelski, A. *Vib. Spectrosc.* **2005**, *39*, 200–213.
